# Leveraging donor support to develop a national antimicrobial resistance policy and action plan: Ghana’s success story

**DOI:** 10.4102/ajlm.v7i2.825

**Published:** 2018-12-06

**Authors:** Japheth A. Opintan

**Affiliations:** 1 Department of Medical Microbiology, University of Ghana, Accra, Ghana

## Abstract

**Background:**

To mitigate the increasing trend of antimicrobial drug resistance (AMR), the Global Action Plan (GAP) on AMR was adopted at the 68th World Health Assembly in May 2015. Subsequently, member countries were encouraged to mirror the five key strategic objectives of GAP to develop their respective National Action Plans (NAPs) by 2017. Country-specific data on AMR is, however, critical for a comprehensive NAP that will inform policy and also anchor all the objectives of GAP. Systematic reviews have been suggested by some authors to generate relevant data to inform NAP development.

**Objectives:**

This article highlights Ghana’s success story in the development of its AMR policy documents and how it could further be implemented through donor support.

**Methods:**

Literature and desk review of the activities of Ghana’s National Platform on Antimicrobial Resistance leading to the development of the NAP and AMR policy was done.

**Results:**

Ghana launched its NAP together with the accompanying policy document in April 2018. Country-specific data, which guided these documents, were obtained by leveraging donor support activities through the National Platform on Antimicrobial Resistance.

**Conclusion:**

Ghana’s success story on the development of AMR policy documents is pivoted on a strong political will and the leveraging of donor support for specific activities.

## Introduction

Antimicrobials are important medicines that revolutionised modern medicine and prevented needless morbidity and mortality associated with infectious diseases. Antimicrobials also paved the way for complex surgical procedures like hipbone replacement to be more possible. Inappropriate use and substandard antimicrobials in humans, animals and aquaculture have, however, contributed to the current global threat of antimicrobial resistance (AMR). To address AMR at the global level, different stakeholders were tasked to put a Global Action Plan (GAP) together. In May 2015, the World Health Assembly adopted GAP’s five key strategic plans to be mirrored by member countries.

The tripartite body, consisting of World Health Organization (WHO), Food and Agriculture Organization (FAO) and International Organization for Animal Health, commissioned and continues to assist several countries to generate the necessary data for individual country National Action Plans (NAPs). Funding from these agencies has led to the development of several assessment toolkits that target laboratory needs, capacities and data generation on AMR.^1^ Some of these toolkits can be applied at the local and country levels. The African Union, through the Africa Centres for Disease Control and Prevention (Africa CDC), recently met in Addis Ababa to discuss a framework for AMR containment within the sub-region. With its audience, authority and access to varied resources, the African Union can better leverage resources for AMR containment in the sub-region. For example, the African Union can effectively complement strategies to control and contain antimicrobial use (AMU) and AMR by formulating the necessary regulatory and legislative frameworks for adaptation by member states.

Inevitably, individual country NAPs, which outline surveillance systems to feed data into global AMU or AMR databases, must start at local and regional levels. At each of these levels, both human and material resources must be adequately leveraged through partnerships. Ghana has been successful at leveraging both internal and donor partners’ resources to develop its NAP and its accompanying policy document.^2,3^ On 11 April 2018, His Excellency, President Nana Addo Dankwa Akufo-Addo, launched both documents which received massive media coverage. This article highlights Ghana’s success story on the development of both documents and also provides some impetus on how further resources can be leveraged for implementation, using the One Health concept.

## Methods

This article is based on literature and desk review of the activities of Ghana’s National Platform on Antimicrobial Resistance (NPAR). The Ministry of Health leads the NPAR which operates on a One Health framework. NPAR works in consultation with public and private agencies, academic and research institutions, professional bodies and civil society organisations. Smaller technical working groups are occasionally tasked to perform various activities at specific time periods and report back to the larger platform.

Financial and technical support for NPAR were provided by the government of Ghana, the Swedish International Development Agency, United Kingdom Department for International Development, the Antibiotic Drug use, Monitoring and Evaluation of Resistance (ADMER) Project, WHO and FAO.

## Coordination and alignment of activities

Leadership is key to the coordination and alignment of all activities that generate data to inform AMR policy. Ghana’s success story began with the formation of the NPAR in 2011. The Director of Pharmacy who led several AMR advocacies^4^ chaired the quarterly meetings of NPAR. The leadership role played by the Ministry of Health and donor support from Swedish International Development Agency through Action on Antibiotic Resistance (ReAct) paved the way for Ghana’s AMR policy.

The NPAR brought together different stakeholders from specialties such as veterinary, research institutions, academia, media and civil society organisations. Bringing different players from human, animal and environmental sectors together meant that individuals who used to work in ‘silos’ began talking to each other and sharing ideas. Sooner, the magnitude and complexities of AMU or AMR became evident to all stakeholders. Ministries like the Ministry of Food and Agriculture, Ministry of Environment, Science, Technology and Innovation, Ministry of Fisheries and Aquaculture Development and the regional FAO eventually joined the crusade. Thus, awareness creation, which forms part of the strategic objectives of GAP, started with discussions around the table.

Through ReAct, the Ministry of Health leveraged support for stakeholder analysis for AMR policy in Ghana. Ministry of Health also worked with civil society organsations to educate the community on issues related to AMU or AMR. Donor support was also leveraged from the ADMER Project, a research project supported by the Danish International Development Agency. The overarching aim of the ADMER Project was ‘prudent use of antibiotics for improved human health’. To inform policy, the ADMER Project constituted an advisory board with membership from key stakeholders, including the Ministry of Health. The chairperson of NPAR leveraged support from the ADMER Project for research activities that fed into Ghana’s AMR policy development.^5,6,7,8^ Ghana-Dutch research activities through the MOH in mid-2005 also provided useful data that covered multiple disciplines. Findings from these research activities provided data for AMR surveillance in humans.^9^ The Newman et al. study was one of the first large-scale studies on antimicrobial drug resistance in humans in Ghana.

The five key strategic pillars of the GAP are anchored on awareness creation, surveillance, infection prevention and control, antibiotic stewardship, and research and innovation. Ghana’s success story is highlighted under these broad strategic objectives below.

## Awareness creation

A knowledge, attitudes, practices and perceptions study was carried out by some members of Ghana’s NPAR in 2014.^10^ Findings from this study showed that there is a gap in the knowledge and perception of optimal antibiotic prescription practices among prescribers. This knowledge gap is not so different from surveys done by WHO in other sub-regions.^11^ Policy and regulatory studies were also leveraged through the ADMER Project.^12^ Importantly, findings from most of the above studies were shared with multiple stakeholders, including sector ministries, directors, policymakers and the general public.

ReAct trained and used civil society organisations to create awareness on AMU or AMR in the communities. Several groups, including opinion leaders, farmers groups and associations, were targeted and sensitised on issues related to AMU or AMR. Since the inception of ‘Antibiotics Awareness Week’, Ghana has been participating, with the Ministry of Health leading various activities including media engagements during these celebrations.

In 2015, ADMER hosted the first ever African Conference on AMR in Ghana with the theme, ‘Who is winning the antibiotic resistance war: Bacteria or man?’^13^ This conference may have created awareness on the largest scale in Ghana and probably the sub-region. The conference was organised in partnership with Ministry of Health, WHO, the University of Ghana and other key stakeholders. Over 120 participants, including health professionals, journalists and the general public, attended the conference.

## Surveillance

Local and regional baseline data is needed to inform AMR policy at country levels. Through Ghana-Dutch collaboration, the first national surveillance of AMR in humans was done in 2007.^9^ In 2015, donor support from ReAct and ADMER was leveraged for a second nation-wide AMR surveillance in Ghana.^8^ The 2015 nation-wide study used WHONET to store and to analyse surveillance data. In addition to the baseline data generated to inform policy, this study identified gaps that needed to be addressed before Ghana’s NAP implementation. For example, human resources, laboratory supplies and quality control checks would be crucial during implementation of a NAP. Compared to human studies, there is a paucity of surveillance data in the veterinary and aquaculture sectors in Ghana. Information on the role of the environment is also lacking. A One Health concept will require building capacities in these other important sectors.

Empirically, it had been suspected that the quality of antimicrobials in the country may not be good, but there were no scientific data to support this fact. However, an important subsequent scientific study by one of the ADMER doctoral students provided some baseline data on the quality of antibiotics in the country. The study showed that most antibiotics purchased from the open market were of poor quality.

## Infection prevention and control

Ghana has a national policy on Infection Prevention and Control (IPC).^14^ However, in practice, the dictates of this policy are not strictly followed. Studies conducted at both tertiary and secondary levels of health care facilities in Ghana, indicate that uptake of IPC practices are not optimal.^12,15^ Small-scale studies in Ghana have shown that compliance to hand hygiene recommendations before and after patient contact are low.^16^ Donor support to improve Water, Sanitation, and Hygiene (WASH) programmes, running water infrastructure and hospital waste disposal are much needed to control infections in Ghana. In many health care facilities in Ghana, the tasks of IPC have been handed to quality assurance committees. This does not augur well for the appropriate implementation of IPC practices. Donor support can be leveraged to train many more full-time IPC personnel.

In 2000, Newman et al., funded by the Neo Pharmacy Centre, conducted a point prevalence survey at the Korle-Bu Teaching Hospital.^17^ This survey gave a prevalence rate of healthcare-associated infections of approximately 7%. Further to this, a team of researchers with funding from the Danish International Development Agency recently conducted a multi-centre point prevalence survey of health care associated infections under the Healthcare Associated Infections Ghana project.^18^ This study was conducted in 10 different hospitals within Ghana, including teaching, regional and district hospitals. The overall prevalence rate was 8% with levels varying between 3.5% and 14.4% among different hospitals.^19^ In addition to this study, doctoral and post-doctoral students on the project are carrying out several other studies on infection control related topics. These include surgical site infections, neonatal and puerperal sepsis, cost associated with health care associated infections as well as ethnography studies.^18^

## Antibiotic stewardship

There is a systematic challenge with antibiotic stewardship in Ghana. There have been reports of high prevalence of unwarranted antibiotic use in health care facilities at all levels of health care.^5,15^ There is often a challenge between excess and access.^20^ The ADMER Project generated some baseline data on antibiotic prescription practices and what informs prescription.^5,12^

## Innovation and research

Local capacity for manufacturing of antibiotics and diagnostics is limited. For example, over 70% of the essential medicine needs of Ghana are imported.^21^ This gives room for the importation of counterfeit and substandard medicines. Donor support and investment is needed to improve local capacity in research, development and production of medicines in Ghana. Studies have shown that several exotic plants in Ghana have medicinal properties. Partnerships with the Centre for Scientific Research into Plant Medicine, Mampong, Akwapim, Ghana could serve a good purpose. There is also the need to leverage donor support for diagnostics, especially point of care tools.^22^

## Perspectives on the implementation of Ghana’s national action plan

The president of Ghana, His Excellency, Nana Addo Dankwa Akufo-Addo, launched both Ghana’s NAP and Policy documents on 11 April 2018. Honorable ministers of state – Ministry of Health, Ministry of Food and Agriculture, Ministry of Environment, Science and Innovation, Ministry of Fisheries and Aquaculture Development – were present to show their commitment. Also present to show their commitment were the tripartite body (WHO, International Organization for Animal Health, FAO), legislators and diplomatic corps. Great leadership from these important statesmen will be required for an effective implementation of the NAP. The implementation target, outlined in the NAP, is between 2017 and 2021. This means Ghana may be late in some targets, although some activities have already commenced.

It is estimated that 21 million dollars will be needed to implement the five-year activities outlined in Ghana’s NAP. Identified lead implementing government ministries and departments would have to factor these activities into their annual budgets. Donor support must also be leveraged for some key activities outlined in the NAP. Fortunately, key activities of the NAP have been costed and lead implementing agencies also identified. This should make it easier for developmental donors to partner with government for the NAPs implementation.

The United Kingdom government’s support through the Fleming Fund is perhaps a low-hanging-fruit that Ghana can fall on, especially for AMU or AMR surveillance in humans, animals, aquaculture and the environment. Ghana can also leverage support from the Fleming Fund to build capacity and infrastructure for the other key strategic objectives including IPC and antibiotic stewardship.

The concept of Ghana’s NAP is One Health. However, greater infrastructure would be required in the animal, aquaculture and environmental sectors. Great political will and AMR champions in these sectors would be required for a successful implementation.

## Conclusions

Through great leadership and donor support leveraging, requisite data was generated to inform Ghana’s NAP and policy documents. A much greater coordination and alignment of activities is required for full and smooth implementation. Donor support can be harnessed through bodies such as the WHO, FAO and the International Organization for Animal Health, the African Union and the Fleming Fund to facilitate implementation.
